# The Impact of Coil Position and Number on Wireless System Performance for Electric Vehicle Recharging

**DOI:** 10.3390/s21134343

**Published:** 2021-06-25

**Authors:** Naoui Mohamed, Flah Aymen, Zaafouri Issam, Mohit Bajaj, Sherif S. M. Ghoneim, Mahrous Ahmed

**Affiliations:** 1 Processes, Energy, Environment and Electrical Systems (Code: LR18ES34), National Engineering School of Gabès, University of Gabès, Gabès 6072, Tunisia; mednaouiing@yahoo.com (N.M.); isam2zaafouri@gmail.com (Z.I.); 2 National Institute of Technology, Delhi 110040, India; mohitbajaj@nitdelhi.ac.in; 3 Department of Electrical Engineering, College of Engineering, Taif University, Taif 21944, Saudi Arabia; s.ghoneim@tu.edu.sa (S.S.M.G.); m.elsamman@tu.edu.sa (M.A.)

**Keywords:** wireless recharge, electrified transportation, magnet flux, Ansys Maxwell software, Coil’s position, electric vehicle

## Abstract

Recently, most transportation systems have used an integrated electrical machine in their traction scheme, resulting in a hybrid electrified vehicle. As a result, an energy source is required to provide the necessary electric power to this traction portion. However, this cannot be efficient without a reliable recharging method and a practical solution. This study discusses the wireless recharge solutions and tests the system’s effectiveness under various external and internal conditions. Moreover, the Maxwell tool is used in this research to provide a complete examination of the coils’ position, size, number, and magnetic flux evolution when the coils are translated. In addition, the mutual inductance for each of these positions is computed to determine the ideal conditions for employing the wireless recharge tool for every charging application. A thorough mathematical analysis is also presented, and the findings clearly demonstrate the relationship between the magnet flux and the various external conditions employed in this investigation.

## 1. Introduction

The area of electric vehicles (EVs) is becoming increasingly relevant in this decade. The majority of transportation applications are electrified and employ an electrical powertrain to supply the necessary mechanical power to these tools [1]. This equipment is often built around an electrical machine linked to an electronic converter that regulates the motor speed dependent on the acceleration requirement [2]. There is also a power pack battery within, which serves as the primary energy source in the event of a pure electric car. This principle energy source must be refilled whenever it is exhausted. Actually, the discharging curve is determined by various factors, including the acceleration form utilized, the battery’s lifetime, the charging/discharging cycle, the battery temperature, and the initial state of charge when the battery is charged [3,4]. Thus, the major of researchers try finding useful solutions for improving the battery lifetime by minimizing the recharge/discharge number or increasing the vehicle autonomy by reducing the quantity of the consumed power from the main energy source, especially by making the recharge action easier. This goal was to assist in developing a wireless power transfer (WPT) technology that might be used to charge an electric vehicle. In this field, the research was not stopped, and more than applications and solutions were presented to improve the performances of this kind of recharge tool [5].

Basing on the conductors’ feedback and the electric car manufacturer’s trends, which is based on the utility and the simplicity of the recharge tool use and how this recharge solution can help increasing selling electric vehicle into the world, the WPT principle can be a useful solution [6,7]. But this technology must be more robust and must have a better energetic rentability. The conventional solution is based on only one receiver placed undo the vehicle, and this prototype does not give enough power. This is conducted to search how it is possible integrating two receivers to undo the car and test the efficiency of this solution. This approach cannot be used until testing is performed to determine how the magnetic field influences the two linked coils and what parameters can impact the entire system.

The leading technology of the wireless energy transmission between a primary and a secondary coil was firstly proposed by Kurs [8]. This system was found suitable and applicable for electric vehicle recharge applications [9]. Basically, wireless charging was applied for stationary cases, and its efficiency was proved. However, the quantity of transferred energy depends on the coil’s size and inverter performances [10,11]. The coil to coil case was tested in more than a technical review, and the only relation between the number of inductance turns is studied for searching about the efficiency [12]. From the other side, this system was studied by working on the different circuit topologies that can be used to affect the system performances [13]. These studies were searched to improve the versions of this WPT technology, but the possibility of using two receivers’ coils was not tested and mainly studied. Some researchers have investigated the use of more than coil applications for some Biomedical applications [14]. The results showed the importance of this addition and proved some related problems related to the resonance between the coils. This is also discussed by the authors of [15,16].

Moreover, working on the coils form was a severe challenge for researchers. The major of the existing literature was used the rectangular coil to form the main structure, due to its immense power. However, placing two closed receiver coils was found difficult in these versions [17,18,19,20]. Actually, the measurement tool of these systems is not very easy, and it needs an initial check for the given performances before starting use.

Based on the previous information and by showing the different works of literature, the wireless recharge system has several weaknesses in relation to the coils centralization and then appeared problem in relation to the recharge time or the power losses. Moreover, the standard version, which bases on only one receiver and only one transmitter, has shown a fewer performance in relation to the recharge time for the electrical transportation system, as the electric vehicle. Therefore, studying the possibility of having two coils receiver under an electric vehicle seems essential to test the actual efficiency of this kind of wireless recharge upgrade. Furthermore, as the placement of two coils in serial, can present a problem regarding the mutual flux, the energetic global performances will be affected. This study gives a detailed analysis and discussion regarding these problems and shows what kind of problem can appear. Finally, it recommends using this upgraded version of the wireless recharge solution.

This paper provides a comprehensive analysis of coil placement in space and demonstrates what benefits may be obtained if more than one receiver coil is utilized for the same transmitter. This study employed the Maxwell tool to display the magnet flux inside and around the coil and the potentially usable space for energy feedback. The forms of flux vectors from the transmitter to the receivers are shown and explained. This gives information about the movement of flux in the field around the coils. At the end of this analysis, the reader will find useful information for correctly modeling the wireless transmitter and have information about the exigence for using a double coil receiver to face the only one coil receiver. The statics of the utility of the double receiver coils face the only one receiver coil on the electric vehicle autonomy is shown at the end and show the weakness and advantages of each proposal.

For having the paper goal, the manuscript is organized as follows. After the introduction section, a general paragraph is implemented, which describes the relationship between the electric vehicle and the wireless recharge method. In the third section, the overall architecture of the principle of the magnetic recharge system is presented. This is by showing the different possible topologies. The fourth section shows the mathematical model of the induction charger system and explains the equations used to quantify the final outputted power. The fifth section shows the main objective of this paper. It contains all the analysis results made on the positions of the coils and dimensions. It also contains the needed information about the practice application, which validates the analysis study made with the Maxwell tool. In the end, the conclusion section concludes the paper by resuming the continent and showing future endeavors.

## 2. Electric Vehicle (EV), Hybrid Electric Vehicle (HEV), and Wireless Recharge Architectures

### 2.1. Hybrid and Pure EV in the Literature

**Figure 1:**
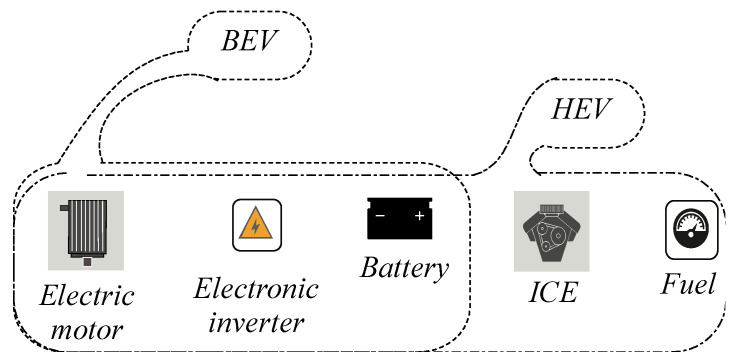
The architecture of the EV and the HEV.

Basically, two models of electric vehicle exit. The hybrid version and the pure version. Figure 1, exposes these two different architectures. The combustion engine is the only different block from the pure electric vehicle prototype in the hybrid version. It can be placed in serial or in parallel with the traction system. Thus, the nomination of a parallel or a serial HEV appears [21].

Basically, inside an electric vehicle, the initial pack of components regroups a source of energy beside a battery system connected to the power electronic converter for feeding the main electric motor. The power managing principle is based on a control loop that is placed into a high-speed calculator, which controls all blocks inside the vehicle [22].

Generally, each of these versions has weaknesses and advantages, and this can be shown in this study [23]. But the main advantage of the pure electric vehicle is the environmental benefit and the absence of any gas emission problem. That advantage has encouraged researchers to make this transport solution more efficient, and many applications, modifications, and contributions have appeared. For example, some researchers try optimizing the size of the traction part, improving the battery technologies, or finding more recharge solutions [24]. 

### 2.2. The Wireless Charging System

**Figure 2:**
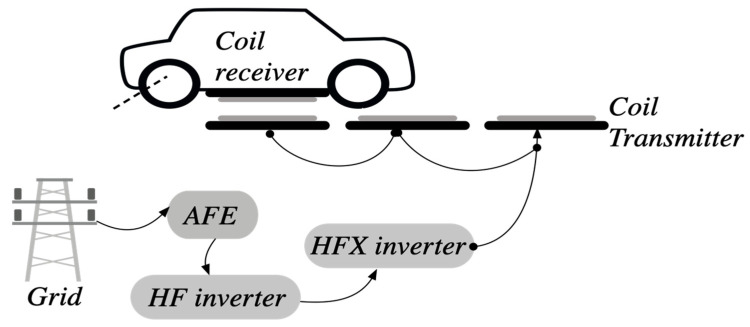
Different needed blocs for the overall WPT system.

As the wireless recharge solution is found suitable for an electric vehicle application, studying correctly, this system is found useful for knowing the weaknesses points and what kind of parameters and variables can affect the rentability. Initially, it is mandatory to know about the overall wireless recharge system, which composes several blocks and has an exceptional architecture. Figure 2 shows these blocs. Basically, a wireless charging system regroups one coil receiver and one coil transmitter. The transmitter can be found in motion for various speeds and positions, and the distance between the two coils is separated by a vacuum, which can be variable. The transmitter block generates alternating magnetic flux with high frequency (HF). This inverter contains a filter bloc adaptable for the high frequency outputted signal, called HFX inverter. This magnetic flux is coupled to the receiving coil and converted into electrical energy that can be used to charge a battery of EV. 

Generally, the basic variable that must be supervised and used for evaluating the performance of the wireless recharge solution. Performance is related to the given electrical power used for charging the battery pack. This depends on the distance between the transmit coil and the receive coil, the amount of energy emitted, according to the positions of the coils [25].

## 3. Different Topologies

For this study, the main objective of the wireless charging system is related to vehicle charging. The recharge action was found depends on various conditions, as the battery state of charge and the type of compensation topology. There are four resonant circuit topologies that could be used in the Induction Power Transfer system. They are named after the way of inserting the resonance capacitors on each side: connection in parallel (P) and/or in series (S). Therefore, the topologies can be described as follows: SS, SP, PS, and PP. Table 1 regroups all of these topologies [26,27]. The corresponding capacitor formula in the receiver or the transmitter coils can be found differences in the compensation topologies. Table 1, gives all these formulas.

## 4. Modeling of the Wireless Charging System: One or Two Coil Receivers 

### 4.1. Wireless Charging System with a Simple Receiver Coil

**Figure 3:**
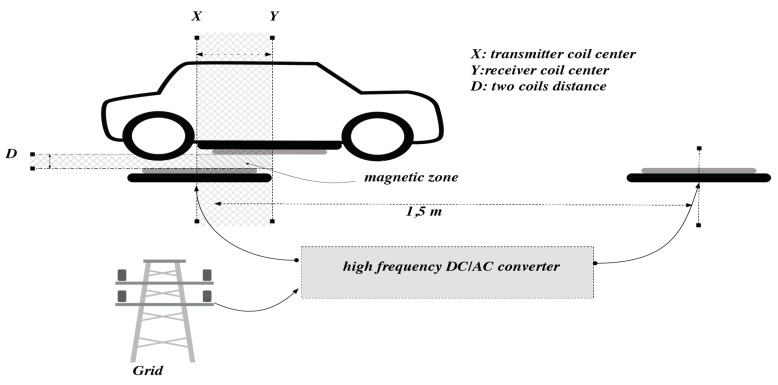
Structure of the inductive power transfer system.

For the electric vehicle application, the wireless charging system is one of the main processes that transfer the electrical energy to the battery to help to charge and increasing vehicle autonomy. Therefore, the study of this system needs to define all possible parameters and variables that can affect the global performances of this recharge tool. Figure 3 illustrates all of these external variables related to the vehicle position face the transmitter coil position. In addition, it shows the case of only one receiver implemented under the vehicle.

**Figure 4:**
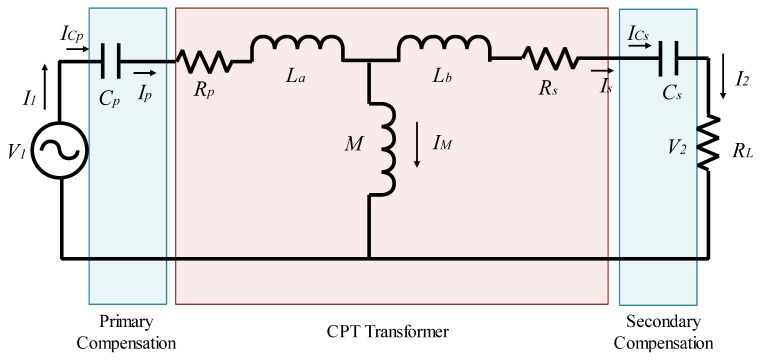
SS compensation topology design with R_L_ load.

In this phase, the selected compensation topology is the SS model, and then the corresponding model is given, as shown in Figure 4. This is by taken into consideration all variable parameters like resistance, local inductances, and mutual inductance. We note by V_1_ and V_2_ the input and output voltages of this IPT [13].

Firstly, it was indicated that $L_{a}=L_{p}-M$, and $\;L_{b}=L_{s}-M$. $L_{a}$, and $L_{b}$ represent the primary and secondary winding leakage inductances, respectively. In this model, the primary voltage of the coil is noted $V_{p}$, which is formed by a sinusoidal voltage source noted *V*_1_. Here, $R_{L}$ represents a serial resistive load. It is used to quantify the final expression of the global yield value, as shown in Equation (7). Expression (1) shows the power consumption formula if a resistive load is connected, considering the mutual inductance parameter in the function of the primary current [28].

(1) $$P_{wr}=I_{p}^{2}.\left(\frac{{\left(\omega M\right)}^{2}}{R_{L}}\right)$$

The global impedance of the primary coil can be expressed in Equations (2) and (3). Thus, the corresponding primary current can be evaluated, as seen in Equation (4).

(2) $$Z_{1}=X+Y$$

(3) $$\{\begin{array}{c} X=\frac{{\left(M\omega \right)}^{2}}{j\omega \left(L_{b}+M\right)-\frac{j}{\omega \cdot C_{s}}+R_{s}+R_{L}}\\ Y=j\omega \left(L_{a}+M\right)-\frac{j}{\omega \cdot C_{p}}+R_{p} \end{array}$$

(4) $$I_{1}=\frac{V_{1}}{Z_{1}}$$

The primary and secondary capacitance dimension noted *C_p_* or *C_s_* must be evaluated under a null imaginary part of *Z*_1_. The corresponding equation of the related capacitance can then be expressed, as seen in Equation (5).

(5) $$C_{p}=C_{S}=\frac{1}{\omega^{2}\left(L_{b}+M\right)}$$

The energetic yield of the WPT system can be evaluated if using the Equation (6). The resistance value greatly influences the yield, as they are supposed to be a real load inside the overall system. Therefore, this expression shows these parameters inside [29].

(6) $$\{\begin{array}{c} \eta =\frac{\left|\vec{I_{s}}\right|\cdot R_{L}}{{\left|\vec{I_{p}}\right|}^{2}\cdot R_{p}+{\left|\vec{I_{s}}\right|}^{2}\cdot R_{s}+{\left|\vec{I_{s}}\right|}^{2}\cdot R_{L}}\\ \frac{\left|I_{p}\right|}{\left|I_{s}\right|}=\frac{R_{s}+R_{L}}{\omega \cdot M} \end{array}$$

Finally, it is concluded that only the reciprocal inductance between the primary and the secondary side coils is connected to the system transmission strength. Therefore, reducing variable parameters makes it easier to study the stability of transmission power and the system efficiency given in Equation (7) [10].

(7) $$\eta =\frac{R_{L}}{R_{L}+R_{s}+\left(\frac{R_{p}.{\left(R_{s}+R_{L}\right)}^{2}}{{\left(\omega M\right)}^{2}}\right)}\;$$

RL, in Equation (7), is the secondary side load impedance, R_2_ and R_1_ are the secondary and primary sides’ internal impedance, respectively. When the charging system is definite, the load and the internal impedance are constant. It can be concluded that the system efficiency is only related to mutual inductance between the primary and the secondary side coils. The condition for achieving optimal efficiency can be deduced from Equation (7). The highest possible efficiency can be achieved if $\frac{R_{p}\left(R_{s}+R_{L}\right)}{\omega^{2}M^{2}}$ tends to 0. As a result:

(8) $$\omega \gg \frac{\sqrt{R_{p}\left(R_{s}+R_{L}\right)}}{M}$$

In that case, the theoretical maximum efficiency will be, as seen in Equation (9).

(9) $$\eta_{max}=\frac{R_{L}}{R_{L}+R_{s}}$$

### 4.2. Wireless Charging System with Multiple Receiver Coils

**Figure 5:**
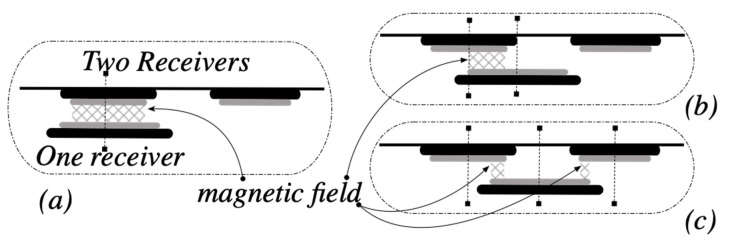
Wireless power transfer design for two receiver coils: (**a**) high magnetic field zone, (**b**) medium magnetic field with one receiver, (**c**) medium magnetic field with two receivers.

According to the present section, to improve the coupling between the coils, a shield can be used to increase the mutual inductance, noted M, by increasing the magnetic flux between the coils and adding another receiver coil. Figure 5 shows the schematic of the secondary side containing two coil receivers (*n* = 2), and it presents how much is the zone of the magnetic field according to the positions of the coils.

The two receiver coils are normally designed identically, and the total impedance reflected from the receiver part is expressed in Equation (10); *n* is the number of receiver coils.

(10) $$\sum_{i=1}^{n}Z_{ri}=n\frac{\omega^{2}M^{2}}{Z_{s}}$$

The equivalent coupling coefficient is then expressed in Equation (11), and this is equivalent to the reflected impedance of a single identical sensor with an equivalent mutual inductance expressed in Equation (12).

(11) $$\{\begin{array}{c} k_{n}=\sqrt{n}\cdot k\\ 0\leq k\leq 1 \end{array}$$

(12) $$M_{n}=\sqrt{n}\cdot M$$

## 5. Impacts of Coil Position and Receiver Coil Number on the WPT Performance

In this section, the performance of the wireless Power Transfer is evaluated according to various external and internal parameters related to the receiver face, the transmitter position, and accordingly to the number of coil receivers. The Ansys Maxwell application is used for applied all of these variables and evaluated the magnetic field value.

### 5.1. Transmitter/Receiver Coil: Design and Parameters

**Figure 6:**
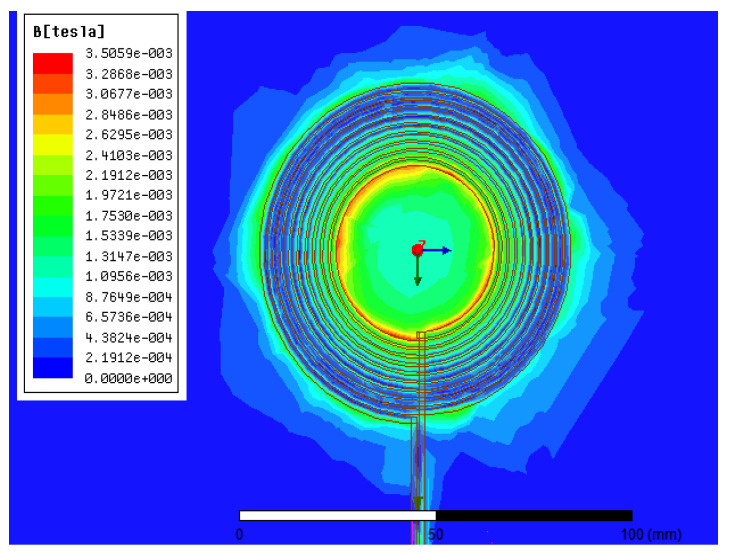
Distribution of magnetic flux density in the coil using Ansys Maxwell software.

Initially, it is mandatory to design correctly the coils that will be used as receivers and/or transmitters. This is must be adapted to the desired application. In this studied case, the design of the coils must be adaptable to be placed undo the vehicle body. Thus, Table 2 regroups all this information and given what is the dimension of the coils [30,31]. Figure 6 also gives the design applied to Maxwell.

After showing these specifications on the coil, it is critical to cite what kind of parameters must be supervised to study the coil coupling factor. For this objective, the coupling coefficient, the mutual inductance, and the coil self-inductance are the factors that will be supervised and discussed. These variables will be studied according to the coil-to-coil relative location, and the coil structural parameters. 

This study needs complicated mathematical analysis work, and this can be guaranteed by a computational calculation method as the Finite element analysis solution (FEA). Thus, using the Ansys Maxwell tool, a variety of solvers for evaluating the electromagnetic part design can be applied. 

**Figure 7:**
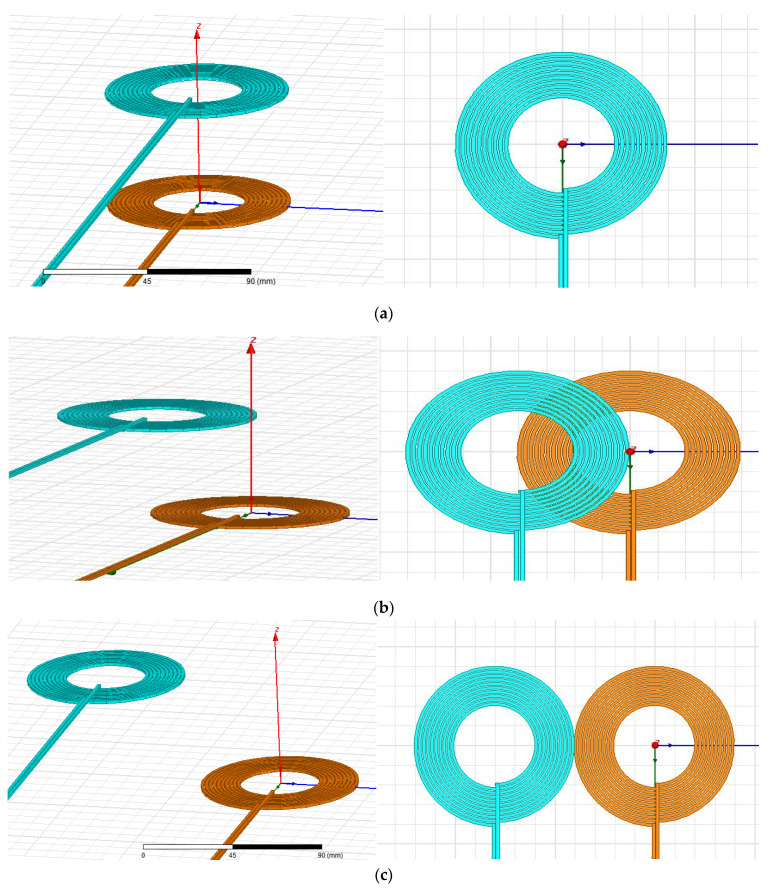
Reference coil model in Ansys Maxwell: (**a**) identical, (**b**) crisscrossed, (**c**) far apart.

With Maxwell’s application, this section tries to study the effect of the variation between the coils in the vertical position. Thus, it appears to the positive or negatively coupling. The results show the analysis of the coupling coefficient and the mutual inductance between the transmitting and receiving coils. The built model can be seen in Figure 7. Figure 6 depicts the distribution of magnetic flux density in the object field, which is a region with a core near the transmitter’s surface. The magnetic field distribution has symmetric properties because the coil structure is strongly symmetrical. Thus, the magnetic flux density near the Litz wire is high in this illustration, while it is low near the central field.

**Figure 8:**
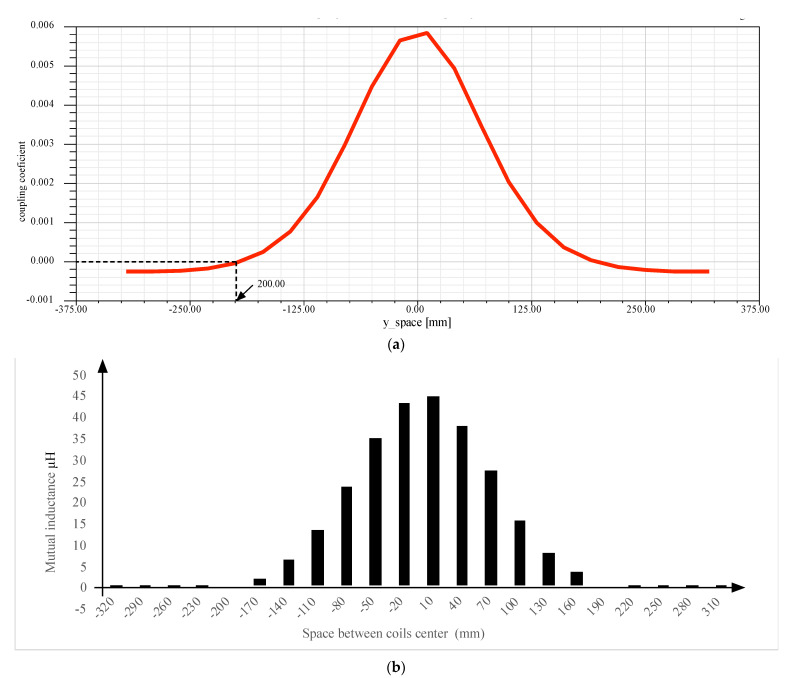
Coil model: (**a**) Coupling coefficient versus the gap variation, (**b**) mutual inductance versus the gap variation.

The primary and the secondary side coil models were designed using the Maxwell tool. The distance between the two-spool axis is variable, and then, the magnetic field simulations were applied for three different cases, as seen in Figure 7. In the first case, Figure 7a, shows the results of the horizontal coil offset, which is equal to 90%. In this study, to simplify the analysis of the system, the WPT consists of two identical circulating coils. Figure 8 shows the three cases of coil pattern circulating for a gap equal to 100 mm.

The simulation of the coreless model was carried out using the Ansys Maxwell software by varying the distance z from 50 to 300 mm.

The overall measurement results of the coupling coefficient and the mutual inductance for the variation of the gap (−250/250 mm) are summarized in Figure 7. The simulations indicate that the value of the coefficient coupling and the mutual inductance decrease as the vertical distance between the transmit and receive coils increases.

This implies that a little power is transferred as the distance between coils increases. A weakly coupled coefficient that characterizes this type of system is less than 0.2.

### 5.2. One Transmitter and One Receiver Coil: Magnetic Field Zones 

The coupling coefficient in Figure 8a shows the influence of the receiver face on the transmitter position. Even the two coils are centralized, and the coupling coefficient is high. This parameter decreases even the two coils center are shifted. At 200 mm the coupling coefficient will be null. When two circuits have a mutual inductance, noted M, it can be said that the two elements are magnetically coupled. This mutual inductance parameter depends on the proper primary and secondary inductances *L_p_* and *L_s_*, respectively. But their relative position affects too. Mathematically, the mutual inductance *M* can be expressed, as seen in Equation (13).

(13) $$M=k\sqrt{L_{p}L_{s}}$$

with *k* the coupling coefficient in Figure 8a. Thus, according to the previous equation, the mutual inductance variation can be shown in Figure 8b. It is clear that the perfect value is always related to the superposition of the two coils element.

**Figure 9:**
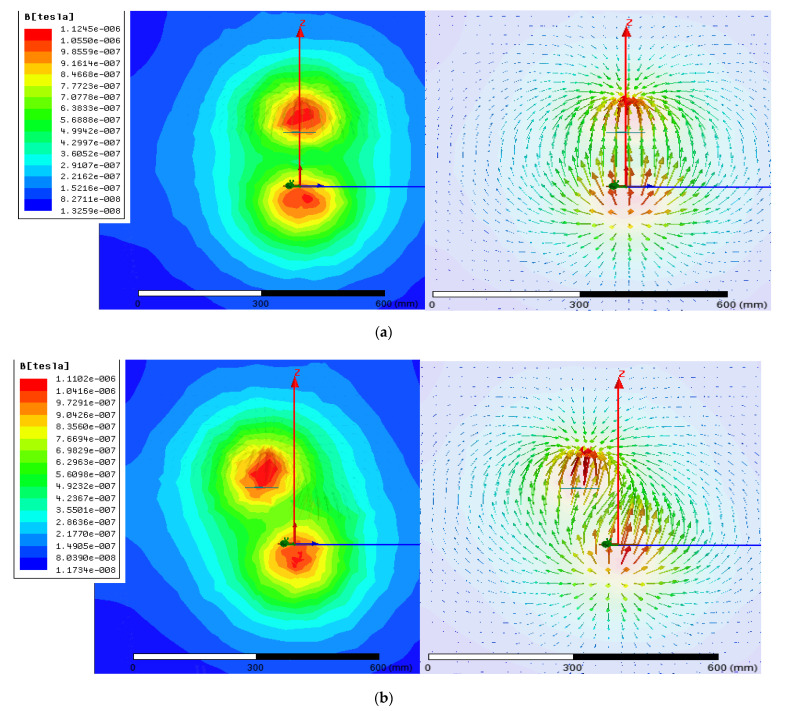
Distribution of the flux density: (**a**) D = 0, (**b**) D = −80.

Figure 9 shows the evolution of the magnetic flux for the case of only one receiver and one transmitter coil. All of the presented screens are taken for two different coil positions. Figure 9a shows that the two coils center are centralized, and it is clear that the magnetic flux is parallel and approximately; all the yields are transferred. On the other hand, the deviation of the magnetic flux is clear for the cases where the two coils are not centralized, as seen in Figure 9b. This situation was depicted for a distance between the two centers equal to 80 mm. Here, the two coils still have a contact zone, as we do not have over-crossed the coil radius. This is why we still have a magnetic zone between the two coils.

### 5.3. One Transmitter and Two Receivers Coils: Magnetic Field Zones 

**Figure 10:**
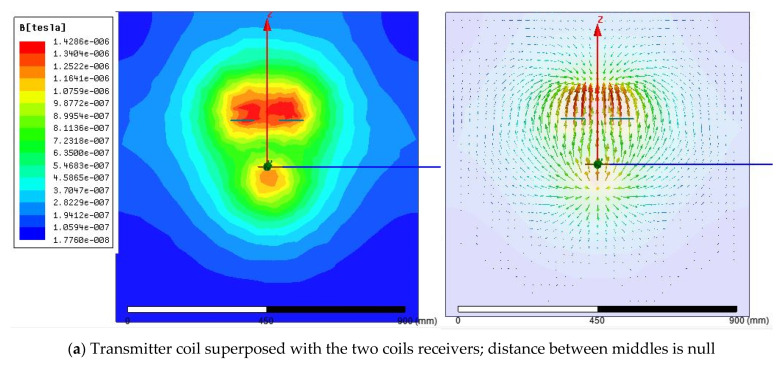

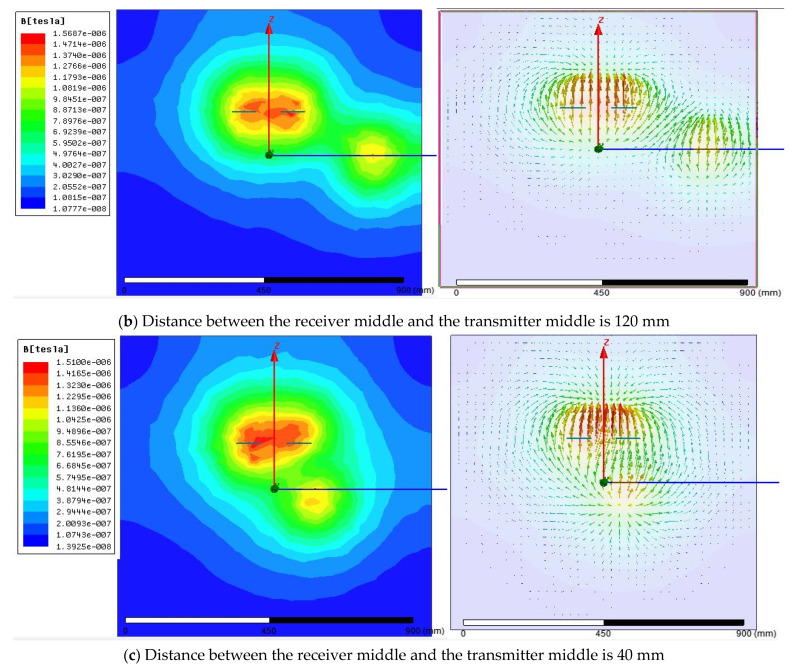
Distribution of magnetic flux for two coils receivers case.

The two-coil receiver system is studied in this section. Moreover, using Maxwell software, the magnetic field evolution is studied, and this is for different cases related to the two-part positions. The built model consists of one coil transmitter, which has the same previous dimensions, cited in Table 2, and the coils receivers, which also have the same performances as the transmitter coil. The distance between the two coils receivers center is 80 mm. As shown in Figure 10a, that the quantity of the magnetic field in the receiver coils is very high as the red color is very concentrated in the centers of the coils. But this is also related to the superposition of the first coil transmitter and two receiver centers. Moreover, it is clear that the mutual magnetic fields are limited between 1.428 tesla as the best value and 1.075 tesla as a minimum value. The form of the magnetic yields is also shown in the same figure, and all yields are parallel.

For the previously cited coil specifications, Figure 10b shows that the transferred magnetic field, cannot be assured as the distance between the two centers is high than the diameter of the one coil receiver. Figure 10b shows the results of the distance between the receiver middle and the transmitter middle is 120 mm, proving why the yields cannot be transferred to the receivers. Moreover, in Figure 10b, the concentration of the red color in the two receivers coils is related to the internal magnetic field that appears between the two receivers coils. However, the color in the transmitter coil is yellow, which means that there is no magnetic field inside. Moreover, the two coils yields do not move from one to the other part; it is just inside the same coil.

For Figure 10c, the case of partial superposition is tested, and this is for 40 mm as applied spacing between the transmitter coil center and the two receivers coils center.

**Figure 11:**
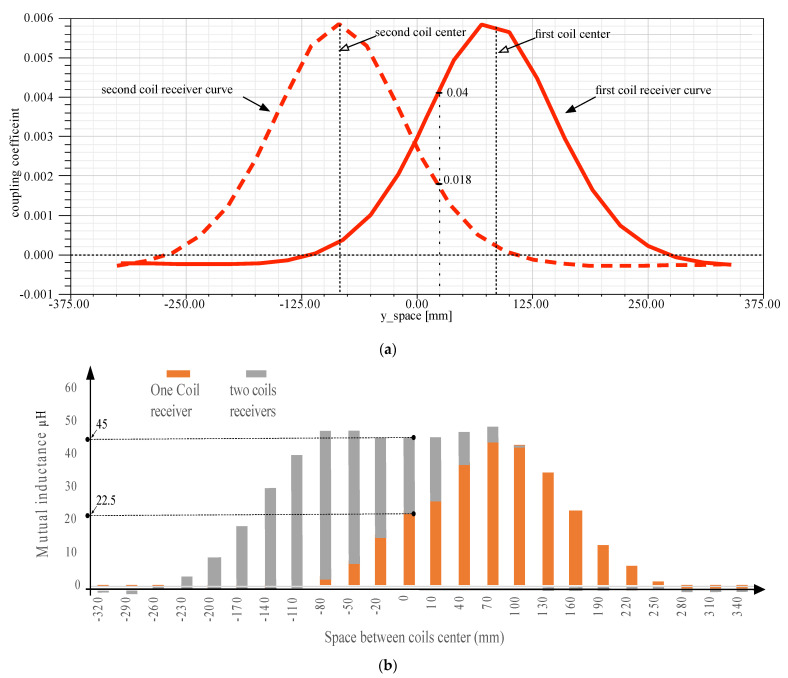
(**a**) Coupling coefficient for each receiver coil proportionally to the transmitter position, (**b**) Mutual inductance for only one receiver coil, and the totally mutual inductance for two receivers coil proportionally to the coil transmitter position.

From the other side, the coupling coefficient for the case of two receivers coils is evaluated, using the given maxwell statistics. Figure 11 shows the corresponding coupling coefficient for each coil proportionally to the transmitter coil position. For example, if the coil transmitter is in the middle of the two receivers coils, the magnetic coupling coefficient can be evaluated as 0.0026 for each coil, and the total will be multiplied by two. However, if the coil transmitter is superposed with the first coil receiver, the maximum coupling coefficient touches 0.0058, and it is the same if it is superposed with the left coil receiver. This is different from the case of only one receiver. It is clear that the range of magnetic coefficient for each coil is the same—but as there are two receivers, the total coupling coefficient will be a sum of two elements. For example, if the two receivers center is spaced from the one transmitter coil center by 0.25mm, then the total coupling coefficient is 0.04 and 0.018. The new total coupling coefficient will increase the efficiency of the WPT.

Figure 11a shows the mutual inductance evolution according to the only one coil receiver and the case of two coil receivers. Proportionally to the interpretation of the previous results, the total mutual inductance will be bigger when the two coils react to the given magnetic field from the transmitter. This is clear for the case, where the transmitter coil center is in the middle of the two receivers coils. Only one receiver coil will have a mutual inductance equal to 22.5 μH, and the same for the second coil—which gives 45 μH as the total mutual inductance. When the two receiver coils move from the center, the given results demonstrate that the high efficiency of the WPT still exists until 80 mm as a marge of variation. When moving more, the efficiency decreases proportionally and touches zero, when the distance is more than 200 mm. As seen in Figure 8b, the totally mutual inductance is better for the case of two coils receivers. For example, for 110 mm as space between the two centers, the mutual inductance is 40 μH for the case of two receivers and 15 μH for the case of one receiver.

On the other hand, this study depicts the possible given power if only one receiver coil is used, and in the case of two receivers, coils were used. Table 3 shows these statistics and resume, the importance of two receiver coils, where it is possible touching 96% of the maximum achieved efficiency. These statistics were depicted for the previously cited coil parameters, as shown in Table 2.

## 6. Experimental Validation

**Figure 12:**
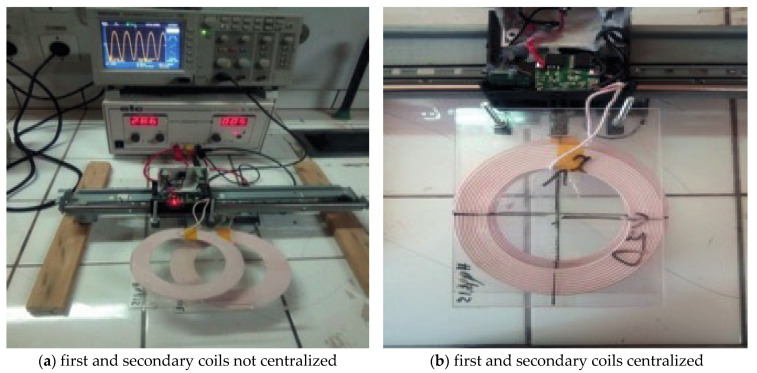
Real prototype for the wireless transfer system.

In this simulation, the prototype operating two coils have two different proper inductances. The first coil has an inductance equal to 30 μH, and the second is 21.13 μH. Figure 12 shows a real photo of the prototype, which the necessary measurement tool. The second, larger, coil is the transmitter part, and the second is the receiver part. In this test, the inputted voltage is equal to 35VDC transforms to an alternating voltage of frequency 50 kHz. The applied test simulates a coil placed undo the vehicle and moved into a different position face the transmitter coil. Moreover, the mutual inductance will be evaluated according to the one or two coils receivers mode. Figure 12 shows this prototype. The real dimensions of the coils are summarized in Table 4. 

**Figure 13:**
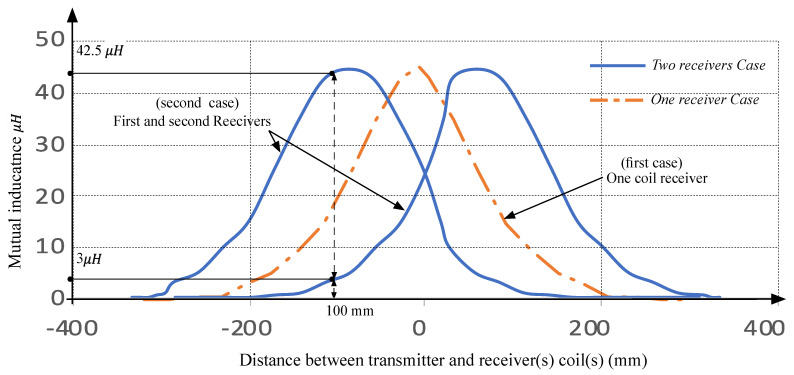
Mutual inductance with one and two receivers.

The given statistics validate the obtained results from the Maxwell application. Thus, if concentrating on the coupling coefficient and the mutual inductance, the importance of the wireless recharge system can be evaluated. For this practical validation, the best mutual inductance value remains constant at approximately 45 μH, until 180 mm as a maximum distance between centers is shown in Figure 13. Whereas, the mutual inductance starts decreasing. Finally, over 200 mm, the mutual inductance comes to zero, and then it is proved that no energy will be transferred to the main charge connected to the receiver coils.

## 7. One and Two Coils Receivers’ Specifications

To show the specifications of the case of two coil receivers face the case of only one coil receiver, Table 5 gives five indication factors that can be used to classify the best receiver combination design. Thus, the classification is made based on: The quantity of the given power, the quantity of the losses power, the receiver coils additive weight in an electric vehicle, the complexity of the electronic converter, and the possible needed time for a full recharge if using each of these architectures.

## 8. Conclusions

At the conclusion of this study, it is feasible to infer that the wireless power transfer system has been investigated. This is by working on the efficiency of two coils receivers face only one receiver. The significance of this investigation is demonstrated by the magnetic fields sent from the first transmitter coil to the case of one or two utilized receivers. The mutual inductance, the coupling coefficient, and the form of the distributed field are supervised according to the transmitter’s translation face the receiver parts. This is the result of the use of the Maxwell tool and proved by a detailed analytical equation. These results were important, and showed how much energy can extract for each position case. Moreover, this study was validated by an experimental prototype that validates the obtained results from the Maxwell application. All aspects of this study, and the efficiency of electric vehicle autonomy, were discussed in the paper.

## Figures and Tables

**Table 1:** Internal parameters of transmitter and receiver coils according to the compensation topology form.

Features	Series-Series (SS)	Series-Parallel (SP)	Parallel-Series (PS)	Parallel-Parallel (PP)
Primary capacitor	$\frac{1}{\omega^{2}\cdot L_{P}}$	$\frac{1}{\omega^{2}\cdot \left(L_{P}-\frac{M^{2}}{L_{s}}\right)}$	$\frac{1}{\omega^{2}\cdot \left(L_{P}-\frac{\omega^{2}\cdot M^{4}}{L_{P}\cdot R_{load}}\right)}$	$\frac{1}{\omega^{2}\cdot \left(\left(L_{P}-\frac{M^{2}}{L_{s}}\right)+\frac{\frac{M^{4}}{L_{s}^{4}}\cdot R_{load}^{2}}{\omega^{2}\cdot \left(L_{P}-\frac{M^{2}}{L_{s}}\right)}\right)}$
Secondary capacitor	$\frac{1}{\omega^{2}\cdot L_{s}}$	$\frac{1}{\omega^{2}\cdot L_{s}}$	$\frac{1}{\omega^{2}\cdot L_{s}}$	$\frac{1}{\omega^{2}\cdot L_{s}}$
Load	$\frac{\omega \cdot L_{s}}{Q_{s}}$	$\omega \cdot L_{s}\cdot Q_{s}$	$\frac{\omega \cdot L_{s}}{Q_{s}}$	$\omega \cdot L_{s}\cdot Q_{s}$

*R_load_* represents the resistance load on the secondary coil.

**Table 2:** Coil used specification on maxwell platform.

Designation	Used Choice
Coil material	Copper
Polygon Segments	4
Polygon Radius	1 mm
Start Helix Radius	20 mm
Radius Change	2.05 mm
Pitch	0
Turns	10
Segments Per Tum	36
Right-Handed	1

**Table 3:** Power transfer factor for the case of one or two receivers coils.

	Distance (mm)	Power Transfer	Efficiency	$Max_{eff}$	$Max_{pow}$	$Av_{pow}$
Simple receiver coil	−200	0.3 kw	10%	92%	4.9 kw	2.34 kw
	−100	1.9 kw	54%			
	−50	3.6 kw	78%			
	0	4.9 kw	92%			
	50	3.5 kw	77%			
	100	1.9 kw	54%			
	200	0.3 kw	10%			
Multiple receiver coils	−300	0.5 kw	12%	96%	5.1 kw	3.95 kw
	−250	3.7	79%			
	−200	4.8	87%			
	−100	4.9	88%			
	−50	5.1 kw	96%			
	0	5.2 kw	96%			
	50	5.2 kw	95%			
	100	5 kw	90%			
	200	4.9 kw	88%			
	250	3.7 kw	79%			
	300	0.5 kw	12%			

$Av_{pow}$: Average outputted power; $Max_{eff}$: Maximum achievable coil system efficiency; $Max_{pow}:$ Maximum achievable output power.

**Table 4:** Real coils dimensions.

Coil diameter	50 cm
Distance between coil	150 cm
Width of winding “w”	21 cm
Average winding radius “r”	14.5 cm
Number of turns “N”	15 Turns

**Table 5:** Specifications of only one receiver coil and two receiver coils.

Specifications	One Coil Receiver	Two Coils Receivers
Maximum Quantity of the given current	140 A	280 A
Quantity of the losses power	10% of the rated total power	20% of the rated total power
Additive weight on the vehicle	+10 kg on the vehicle weight	+20 kg on the vehicle weight
Electronic complexity	medium	high
Needed recharge time in stopped mode *	8 h	6 h
Needed recharge time in motion mode *^,1^	50 h	26 h

*: For 220 volt as input voltage; *^,1^: For a road totally equipped with a wireless transmitter and for 50 km/h as car speed value; All of these statistics were depicted from these references [10,28,32,33,34,35].

## Data Availability

Not applicable.

## Abbreviations

*IPT*	Inductive Power Transfer
*EV*	Electric Vehicles
*HEV*	Hybrid Electric Vehicle
*HF*	High-Frequency
*AFE*	Active Front End
*WPT*	Wireless Power Transfer
*SWC*	Static Wireless Charging system
*FEM*	Finite Element Method
*FEA*	Finite Element Analysis
*M*	Mutual inductance (H)
*ω*	Oscillation angular frequency (rad/sec)
*k*	Coupling coefficient
*L_s_*	Secondary inductance (H)
*L_p_*	Primary inductance (H)
*Z_p_*	Primary impedance (Ω)
*Zs*	Secondary impedance (Ω)
*I_p_*	Primary current (A)
*I_s_*	Secondary current (A)
*V_p_*	Primary voltage (V)
*V_s_*	Second voltage (V)
*I*_1_	Source current (A)
*I*_2_	Load current (A)
*C_s_*	Secondary capacitance (F)
*C_p_*	Primary capacitance (F)
*P_s_*	Secondary power (W)
*P_p_*	Primary Power (W)
*L_a_*	Primary leakage inductance (H)
*L_b_*	Secondary leakage inductance (H)
*R_L_*	Load (Ω)
*η*	The efficiency of the power transfer (%)
*Z*_1_	The global impedance of the primary coil (Ω)
*V*_1_	Source voltage (V)
*R_s_*	Secondary resistance (Ω)
*R_p_*	Primary resistance (Ω)
*n*	Number of receiver coils
*D*	Distance between the receiver middle and the transmitter middle
